# Early Relaxation Dynamics in the LC 13 T Cell Receptor in Reaction to 172 Altered Peptide Ligands: A Molecular Dynamics Simulation Study

**DOI:** 10.1371/journal.pone.0064464

**Published:** 2013-06-06

**Authors:** Bernhard Knapp, Georg Dorffner, Wolfgang Schreiner

**Affiliations:** 1 Center for Medical Statistics, Informatics and Intelligent Systems, Section for Biosimulation and Bioinformatics, Medical University of Vienna, Vienna, Austria; 2 Department of Statistics, Protein Informatics Group, University of Oxford, Oxford, United Kingdom; 3 Center for Medical Statistics, Informatics and Intelligent Systems, Section for Artificial Intelligence, Medical University of Vienna, Vienna, Austria; University of Bologna & Italian Institute of Technology, Italy

## Abstract

The interaction between the T cell receptor and the major histocompatibility complex is one of the most important events in adaptive immunology. Although several different models for the activation process of the T cell via the T cell receptor have been proposed, it could not be shown that a structural mechanism, which discriminates between peptides of different immunogenicity levels, exists within the T cell receptor. In this study, we performed systematic molecular dynamics simulations of 172 closely related altered peptide ligands in the same T cell receptor/major histocompatibility complex system. Statistical evaluations yielded significant differences in the initial relaxation process between sets of peptides at four different immunogenicity levels.

## Introduction

T cells (TC) do not recognize natural antigens but short peptides (called T cell epitopes) which are presented in the context of major histocompatibility complex (MHC) molecules on the surface of specialized antigen presenting cells (APCs) after uptake and processing. The interaction between T cell receptor (TCR), peptide, and MHC (TCRpMHC) is one of the most important processes in adaptive immunology. However, the detailed structural mechanism of T cell activation (TCA) is still unknown [1]. Several models for the mechanisms involved in TCR triggering have been proposed [2]: roughly they can be grouped in aggregation models, models based on conformational changes, and segregation models. It is known that T cells can recognize seemingly dissimilar epitopes [3] where sequence similarity alone is not sufficient to explain immunogenicity [4] suggesting that structural rearrangements [5], changes in heat capacity (which can be an indicator of conformational change or flexibility [6]), biochemical similarities [7], hydrophobicity, molecular weight and structural preferences [8] may play an additional important role in determining immunogenicity. Hence it is difficult to develop predictive methods for peptide immunogenicity and only few methods have been published. For example, POPI [9] and its extension POPISK [8] are based on physicochemical properties. Also the general alignment methods ALIGN [10] and PSI-BLAST [11] were used to align new peptides with known immunogenic ones [9]. In contrast, the binding affinity between peptide and MHC can be predicted with rather high accuracy [12] and therefore many predictive studies use the peptide binding affinity to MHC as an approximation for immunogenicity [13]. However, this does not provide the full picture: while binding affinity (usually <500 nM [14]) is a prerequisite for immunogenicity, the magnitude of the pMHC affinity does not correlate well with the magnitude of immunogenicity ([8] and therein references). The explanation for immunogenicity is rather to be found in a synergistic combination of TCRpMHC affinity, mean interaction time, and relative abundance of both complexes. However, to incorporate all these factors in a (structural) predictive model currently does not seem feasible.

Molecular dynamics (MD) [15] is a computational method to solve Newton’s equations of motion for a given system of atoms. Various MD studies have been performed in relation to TCRpMHC interaction: Cuendet et al. investigated the dissociation of the TCR from the pMHC via a steered MD simulation and gave insight into the dissociation mechanism of two complexes, which differed by only a single amino acid mutation [16]. Yaneva et al. performed MD simulation studies of HLA-DR3 with and without invariant chain-associated peptide (CLIP) and found that larger conformational changes of alpha-helices flanking the MHC binding groove occur without CLIP [17]. Wan et al. performed free energy calculations between pMHC and TCR [18]. Painter et al. [19] as well as Zacharias et al. [20] compared the peptide-bound and non-bound state of the MHC molecule via MD studies. Wan et al. published a large scale MD simulation of the whole TCRpMHC complex and CD4 complex including the surrounding membranes [21]. Rognan et al. studied and predicted the interaction between pMHC and the T cell receptor via molecular dynamics simulation [22]. Haidar et al. performed several in silico point mutations in TCRs to increase the binding affinity to pMHC [23]. Camacho et al. performed a structural and thermodynamic approach to modeling peptide immunogenicity for protein antibodies [24]. De Rosa et al. described a protocol to model the structure of a TCR based on its sequence on a pMHC complex. Subsequently they verified their results via MD simulations [25]. Stavrakoudis perfomed a single MD simulation of the LC13 TCR in complex with HLA-B*08∶01 [26]. Cuendet et al. performed a steered MD study of 3 TCRpMHC complexes [27]. In this study they investigated the dissociation of the TCR on the basis of the reduction of hydrogen bonds, an increase of water in the interface and energetic changes. Narzi et al. used MD simulations to investigate the disease associated MHC alleles HLA-B*27∶09 and HLA-B*27∶05 with different viral and self-peptides [28]. In a previous study our group showed, via a combination of MD simulation, pMHC binding assays, and in vitro T cell activation assays, that an N-terminal peptide flanking region (PFR) of MHC class II can significantly influence the immunogenicity compared to the same peptide without the PFR [29]. Recently we could also show the molecular background of mug pollen Art v 1_25–36_ bound to HLA-DR1 and HLA-DR4 [30]. In another study we compared data of 3 altered peptides ligands (APLs) related to experimental allergic encephalomyelitis [31]. Since it is known that altered peptide ligands often induce alterations in the TCRpMHC interface [32] we extended our approach of investigating APLs via MD to a more systematic screening of 172 well described and strongly related TCRpMHC systems. For this purpose we performed a total of 192 MD simulations with a total length of 2 720 ns and found indications that more and less immunogenic complexes might have slightly different initial relaxation dynamics.

## Methods

### Experimental Data

We selected the protein data bank [33] identification (PDB-id) 1mi5 as a structural basis for our study. It contains the crystal structure of LC13 TCR in combination with HLA-B*08∶01 and the Epstein Barr Virus (EBV) peptide with the amino acid sequence FLRGRAYGL. We chose this system for two reasons. Firstly, the TCRpMHC structure has been determined and described as a whole (PDB-id 1mi5; [34]) as well as in its unliganded parts: TCR (PDB-id 1KGC; [35]) and pMHC (PDB-id 1M05; [36]). Secondly, Kjer-Nielsen et al. depict a systematic substitution study of all 9 amino acid positions in the peptide with the remaining 19 standard amino acids. For each of these mutations they provide the results [34] of a cytotoxicity assay [37] over a range of peptide concentrations. This yields 172 (20+8×19) experimental immunogenicity values for APLs of the same TCR/MHC complex. This data set allows for a systematic and explorative comparison between effects induced by more and less immunogenic peptides.

### Simplifications in the TCRpMHC Structure Avoided

Since the TCRpMHC is a large complex, many authors have only simulated the variable regions of the TCR, the epitope and the α1 and α2 domain for MHC class I, or the α1 and β1 domain for MHC class II. There is evidence that this reduction may be legitimate [38]–[42]. However, this view is not shared by everyone, see, for instance, [43]. In our study the aim is to track subtle changes in shape and dynamics. Hence we simulated the full TCRpMHC without any simplifications in the available TCRpMHC x-ray structure. This includes the constant regions of the TCR as well as the α3 region and β2 microglobulin of the MHC.

### Construction of the Simulated Complexes

We modeled all 172 TCRpMHC complexes on the basis of PDB accession code 1mi5. These 172 complexes differ from each other only by one amino acid substitution in the peptide. We performed these amino acid side chain substitutions with SCWRL [44] and visually confirmed them with the substitution method of SPDBV [45], as this combination turned out to be the most appropriate way [46], [47].

### Molecular Dynamics Workflow

We performed MD simulations using Gromacs 4 [48] according to the following workflow. We immersed each modeled TCRpMHC structure into a three-dimensional explicit water cube with side length of 119 Å, allowing for a minimum distance of 20 Å between protein and box-boundary. Additionally, we applied periodic boundary conditions. Subsequently, we used a steepest descent method to minimize the energy of the system. In the next step we warmed the system up to 310 K. Finally, MD simulations were carried out for a simulation time of 10 ns for each complex (in total yielding a simulation time of 1720 ns) using bond constraints that allowed for an integration step of 3 fs [49], [50]. Further simulation parameters were set to values derived in one of our previous studies [42].

To further investigate the behaviour of the simulations over a longer time period, we additionally performed 50 ns simulations of 20 TCRpMHC complexes. For this purpose we choose the complexes with mutations in position 7 of the peptide. This position is considered as pivotal for the recognition process by the TCR since the tyrosine present in the x-ray structure protrudes deep within a pocket created by CDR1alpha, CDR3alpha, and CDR3beta of the TCR [34]. Together with the 10 ns simulations this yields a total simulation time of 2 720 ns (10×172+20×50).

### Regions of Interest within the TCR

**Figure 1:**
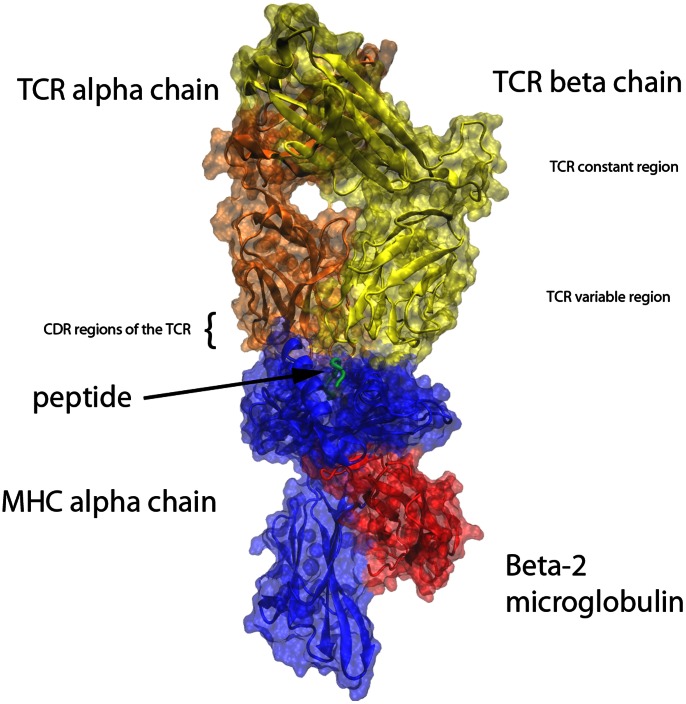
Illustration of the overall TCRpMHC complex. **Blue: MHC alpha chain.** Red: Beta-2 microglobulin. Green: Presented Peptide in the MHC binding groove. Orange: TCR alpha chain. Yellow: TCR beta chain.

The structure of the investigated TCRpMHC complex is shown in Figure 1. To systematically investigate the TCR we grouped all residues according to the secondary structure labelling and complementary determining regions (CDR) labelling from [35], as well as the secondary structure labelling provided by the program VMD [51]. In total we investigated 94 different residue groups (see Table 1 for a detailed list).

**Table 1:** TCR regions of interest according to Kjer-Nielsen et al. and VMD.

region	chain	start residue	end residue	group nr		region	chain	start residue	end residue	group nr
**Chain nomenclature**						**LC13 TCR beta strand nomenclature according to VMD**				
Protein	ABCDE	1	827	1		Constant alpha chain				
TCR alpha	D	386	586	2		a-b loop (Beddoe et al.)	D	509	516	61
TCR beta	E	587	827	3		a	D	504	510	62
TCR	DE	386	827	4		linker a:b	D	511	516	63
						b	D	517	523	64
**LC13 TCR CDRs nomenclature according to Kjer-Nielsen et al.**						linker	D	524	537	65
framework region begin	D	386	406	5		c1	D	538	540	66
CDR1 alpha	D	407	416	6		linker	D	541	543	67
framework region CDR1a:CDR2a	D	417	432	7		c2	D	544	548	68
CDR2 alpha	D	433	438	8		linker	D	549	552	69
framework region CDR2a:CDR3a	D	439	473	9		d	D	553	562	70
CDR3 alpha	D	474	484	10		linker	D	563	568	71
framework region CDR3a:linker	D	485	495	11		e	D	569	571	72
linker V:C	D	496	503	12		linker to transmembran region	D	572	586	73
framework region begin	E	587	608	13		Constant beta chain				
CDR1 beta	E	609	616	14		a	E	707	712	74
framework region CDR1b:CDR2b	E	617	632	15		linker	E	713	714	75
CDR2 beta	E	633	638	16		linker	E	715	721	76
framework region CDR2b:CDR3b	E	639	678	17		linker	E	722	722	77
CDR3 beta	E	679	687	18		b	E	723	733	78
framework region CDR3b:linker	E	688	697	19		linker	E	734	737	79
linker V:C	E	698	706	20		c	E	738	744	80
						linker	E	745	746	81
**LC13 TCR beta strand nomenclature according to Kjer-Nielsen et al.**						d1	E	747	749	82
V alpha chain						linker	E	750	752	83
a1	D	387	388	21		d2	E	753	755	84
linker a1:a2	D	389	391	22		linker	E	756	759	85
a2	D	392	396	23		d3	E	760	761	86
linker a2:b1	D	397	400	24		linker	E	762	770	87
b1	D	401	407	25		e	E	771	780	88
linker b1:c1 (∼ CDR1 alpha)	D	408	415	26		linker	E	781	785	89
c1	D	416	421	27		linker	E	786	789	90
linker c1:c2	D	422	427	28		f	E	790	797	91
c2	D	428	433	29		linker	E	798	815	92
linker c2:c3 (∼ CDR2 alpha)	D	434	437	30		g	E	816	823	93
c3	D	438	439	31		linker to transmembran	E	824	827	94
linker c3:d	D	440	442	32						
d1	D	443	447	33						
linker d:e	D	448	452	34						
e1	D	453	458	35						
linker e:f	D	459	466	36						
f1	D	467	472	37						
linker f:g (∼ CDR3 alpha)	D	473	489	38						
g1	D	490	495	39						
V beta chain										
a1	E	589	591	40						
linker a1:a2	E	592	593	41						
a2	E	594	598	42						
linker a2:b1	E	599	602	43						
b1	E	603	608	44						
linker b1:c1 (∼ CDR 1 beta)	E	609	614	45						
c1	E	615	621	46						
linker c1:c2	E	622	627	47						
c2	E	628	634	48						
linker c2:c3 (∼ CDR 2 beta)	E	635	636	49						
c3	E	637	640	50						
linker c3:d1	E	641	648	51						
d1	E	649	652	52						
linker d1:e1	E	653	658	53						
e1	E	659	663	54						
linker e1:f1	E	664	671	55						
f1	E	672	679	56						
linker f1:g1 (∼ CDR 3 beta)	E	680	686	57						
g1	E	687	688	58						
linker g1:g2	E	689	691	59						
g2	E	692	697	60						

### Root Mean Square Deviation (RMSD) Calculations

The RMSD is defined as
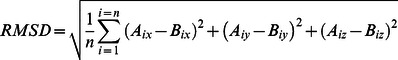
where *n* is the number of atoms, *i* is the current atom, *A* is the target structure and *B* is the reference structure. We employed the *g_rms* function of Gromacs to calculate the backbone RMSD values for the regions of interest described in Table 1. Thereby we used two different fitting strategies for the simulation trajectories. Since we are interested in the deformations of the TCR we firstly fitted the model to pMHC to obtain insight into the overall movements of the TCR and, secondly, we fitted it to the TCR itself to investigate the relative deformations within the TCR. Note that these RMSD calculations yield the norm in Euclidean space between each single time step and a reference structure, which in our case was the configuration at time 0.

### Statistical Evaluation

Although immunogenicity is a continuous variable and not a binary property, we had to create such a binary property, between more and less immunogenic peptides, by segregating the peptides - at four different concentration thresholds - into two classes: the more immunogenic peptides (“groupM”), and the less immunogenic peptides (“groupL”).

- Threshold 1 is determined at the upper limit of the experimental specificity assay. This means that all APLs which were able to induce 50% of maximum lysis at an arbitrary concentration within the range of the assay [34] are classified as groupM. This threshold is determined at 10^–5^ M and yields 121 APLs (or 1210 ns of simulation time) in the groupM and 51 APLs (510 ns) in the groupL.
- Threshold 2 is determined at 10^–6^ M and yields 90 APLs (900 ns) in the groupM and 82 APLs (820 ns) in the groupL.
- Threshold 3 is determined at 10^–7^ M and yields 55 APLs (550 ns) in the groupM and 125 APLs (1250 ns) in the groupL class.
- Threshold 4 is determined at the lower limit of the assay. This means that only the most immunogenic APLs pass this threshold. This threshold is determined at 10^–8^ M and yields 33 APLs (330 ns) in the groupM and 139 APLs (1390 ns) in the groupL.

For each of the 94 regions and each of the 3350 time points (10 ns of simulation time) we performed an unpaired t-test between the RMSD values of the groupM and the groupL TCRpMHC complexes at different significance levels, α. In other words: t-test{[(all “groupM” RMSD values), (all “groupL” RMSD values)] | time, region, α}. This leads to a number of 314900 (3350×94) unpaired t-tests. These are illustrated as a 1-bit image of significances (see result section). We conducted this procedure for the 4 thresholds determined above, both fitting strategies (see previous section) and p-value thresholds at α = 0.05, 0.01, and 0.001, respectively, leading to a total number of 7557600 unpaired t-tests.

It is obvious that p-values of this procedure cannot directly confirm statistical significance on the differences between the two classes due to the large number of tests. Therefore, we merely compute labels indicating whether p<α only, as an intermediate measure for differences. Statistical testing is relegated to one step further. Our new null hypothesis is that there are no consistent differences between groups and thus labels indicating p<α should occur only sporadically and about equally distributed over time and space. The alternative hypothesis is that differences show a systematic structure with a large number of adjacent labels, both in time and space.

We define three measures, applied to the entire map (image), expressing the extent to which the maps show such a systematic structure.

The first such systematicity measure is a reduction of “salt and pepper noise” while the edges of the image are preserved. For this purpose we used a 2-dimensional median filter employing a 3×3 window. This systematicity measure is defined as
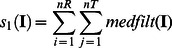
where **I** is the *nR x nT* image matrix of “pixels” (*nR = *number of regions, *nT = *number of time points), with each “pixel” being 1 if p<α in the t-test, 0 otherwise. The term *medFilt* is the median filter as described above. Note, that not all consecutive regions and residues are covalently bound. There are 6 exceptions at the end of the regions which are not adjacent to the beginning of the next region (region numbers 4, 12, 20, 39, 60, and 73). These regions cannot be expected to correlate with their non-adjacent neighbours in the region list of Table 1. Thus, we computed *s_1_* for 7 distinct sub-images of the 94×3350 image and summed up the results. By this way only regions that show consistent adjacent differences in both space and time yield large values of *s_1_*. We also considered the aspect that the application to 7 sub-images may penalize boundary regions of the sub-images too strongly. Hence, we also implemented this method for the image as a whole. However, the results were almost identical (data not shown). Therefore we refer to the median-method as the method being applied to 7 sub-images.

The second, alternative, systematicity measure is defined as
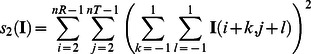



In other words, for each complete 3×3 window the sum of all “pixels” equal to 1 is squared, and the resulting values are summed up. This approach is also applied to the sub-images mentioned above. Thus, large values of *s_2_* are obtained only if adjacent regions show consistent differences in both space and time. This approach is referred to as the square-method.

In the third approach we did not apply any kind of filtering to the image and directly add up the “pixels” equal to 1:
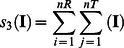



This approach is referred to as the direct-method.

In order to finally test whether an observed systematicity value is significantly high, we approximate the distributions of *s_1_*, *s_2_* and *s_3_* under the null hypothesis “there is no structure” by taking 500 random permutation splits of all APLs for the thresholds 1 to 4 determined above. Note that this yields 500 permutations with 121 against 51 APLs, 500 permutations with 90 against 82 APLs, 500 permutations with 47 against 125 APLs, and 500 permutations with 33 against 139 APLs. These permutations were performed independent from position meaning that the APLs were chosen from any position and were not restricted to the grouping of their initial position in the peptide.

We then calculated the 95% percentile of the respective random distributions as critical values for the values of *s_1_*, *s_2_* and *s_3_* of the true splits. If that value is larger than the critical value then we can speak of a significant structure (in its entirety, not in any detail) and reject the null hypothesis.

## Results and Discussion

### Are Differences to be Found in Local Phenomena Rather than in Global Rearrangements?

**Figure 2:**
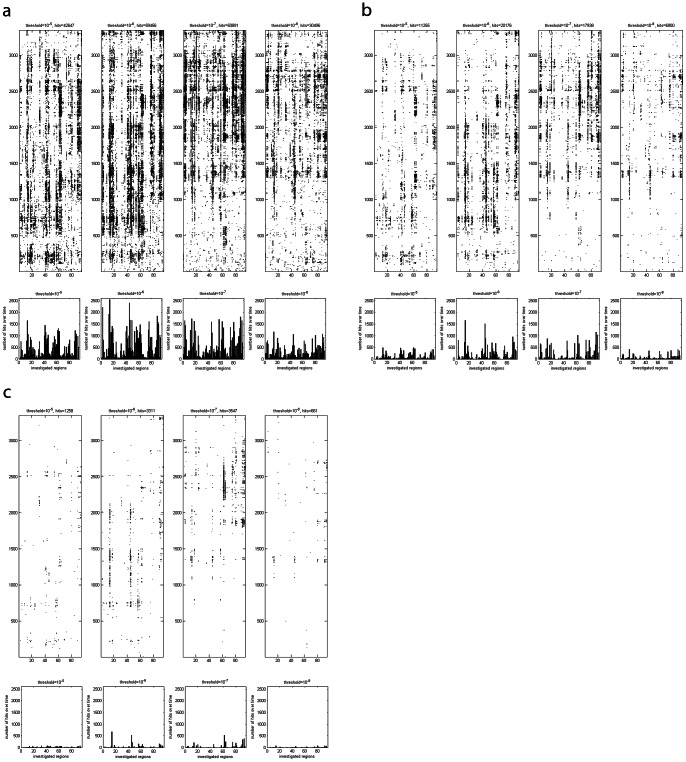
Images of differences, as measured by t-tests. A black dot at time step x and region y indicates that a t-test{[(all “groupM” RMSD values), (all “groupL” RMSD values)] | x, y, α} yielded a difference. In the subplots below the images of differences the total number of hits per region and over time is illustrated. (A) Image of differences for α = 0.05. (B) Image of differences for α = 0.01. (C) Image of differences for α = 0.001.

As described in the methods section we first performed unpaired t-tests and created the corresponding difference images. The first major difference occurs between the 2 fitting strategies. It turns out that the fitting of the simulation trajectory to the TCR leads to a much sharper discrimination between the groups (data not shown) and therefore only this version is discussed in detail. In the TCR fitting version several regions are systematically highlighted as different between the groups (Figure 2). In particular, in several regions continuous “lines” of differences over a longer time period can be observed. This effect of fitting suggests that the spatial rearrangements during the very early relaxation process in reaction to different peptide immunogenicity classes consist of local phenomena within the TCR rather than of global rearrangements of the TCR. This is in agreement with the literature which proposes small shifts instead of major structural changes [32]. However, since the simulation time in MD simulations is limited (10 and 50 ns respectively) major structural changes occurring later cannot be disproven by this study.

### “Lines” of Differences Over Time Remain Visible Even for Smaller Alphas

**Figure 3:**
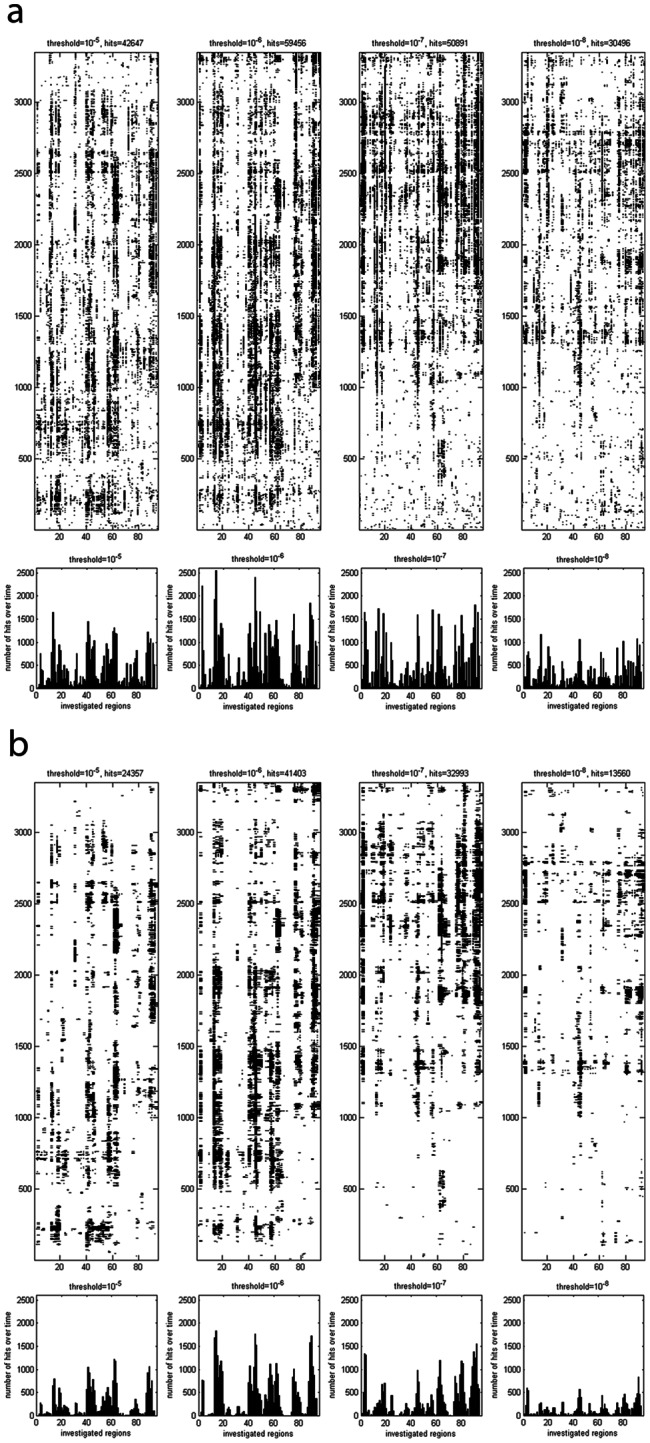
Effect of the median-method. (A) The identical data as in Figure 2A is illustrated. Again a black dot at time step x and region y indicates that a t-test{[(all “groupM” RMSD values), (all “groupL” RMSD values)] | x, y, α} yielded a difference. This approach is termed the direct-method. (B) The identical data as in Figure 2A is illustrated, however, processed with the median-method.

We additionally applied p-value thresholds of α = 0.01 and α = 0.001 (Figure 2) to reduce the number of random hits. Even though the p-value threshold is reduced the rough positions of the “lines” over time remain visually (Figure 2 map plots), as well as in the sum over time for the different regions (Figure 2 bar plots). The same applies for the median-method (Figure 3).

### Significance Testing: Evidence for Significant Difference between the TCRpMHC Groups for the Entirety of Regions

**Figure 4:**
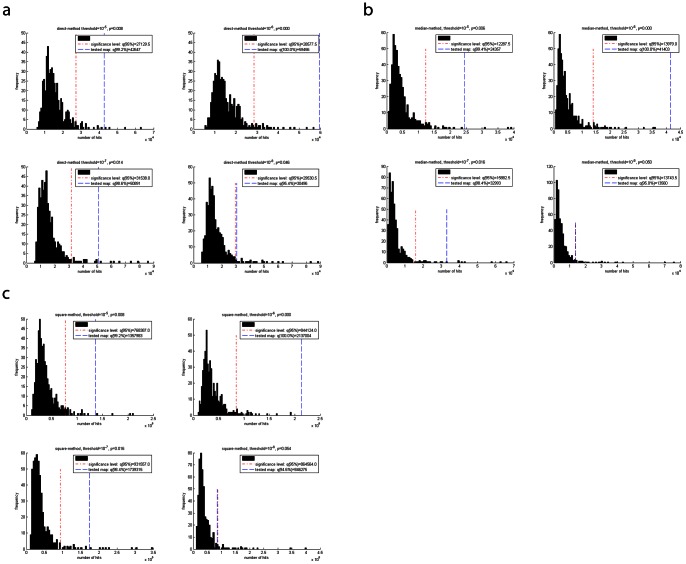
Distribution of s in 500 random permutation splits. The 95% percentile and the tested map (value of s for the true split) are indicated for the four different thresholds per method. (A) Direct-method. (B) Median-method. (C) Square-method.

Following the methods described above in the methods section, we simulated the distribution of systematicity values s by creating 500 random permutation splits between the groups of TCRpMHC complexes. These distributions of the random splits are depicted as histogram in Figure 4, together with the 95% percentiles as critical values. At threshold 1 (10^–5^ M) and using the direct-method only 4 out of 500 random permutations yielded a higher number of hits than the true split (p = 0.008). The results for the square-method were identical. For the median-method the result is marginally better (p = 0.006). At threshold 2 (10^–6^ M) all 3 methods yield the true split as the one with the highest number of hits. Note that this does not necessarily mean that the true split has the most extreme differences of all possible combinations; it means that within these 500 random splits none was more extreme than the true split. At threshold 3 (10^–7^ M) the results for the median and square-method were about equal (p = 0.016). The direct-methods is marginally better in the discrimination (p = 0.014). At threshold 4 (10^–8^ M) the direct-method narrowly reaches statistical significance (p = 0.046) while the median and square-method both narrowly fail the significance level (0.050 and 0.054 respectively). This could be caused by the relatively strong imbalance of 33 versus 139 TCRpMHC complexes for this threshold. However, one could still argue that there is a strong tendency.

**Figure 5:**
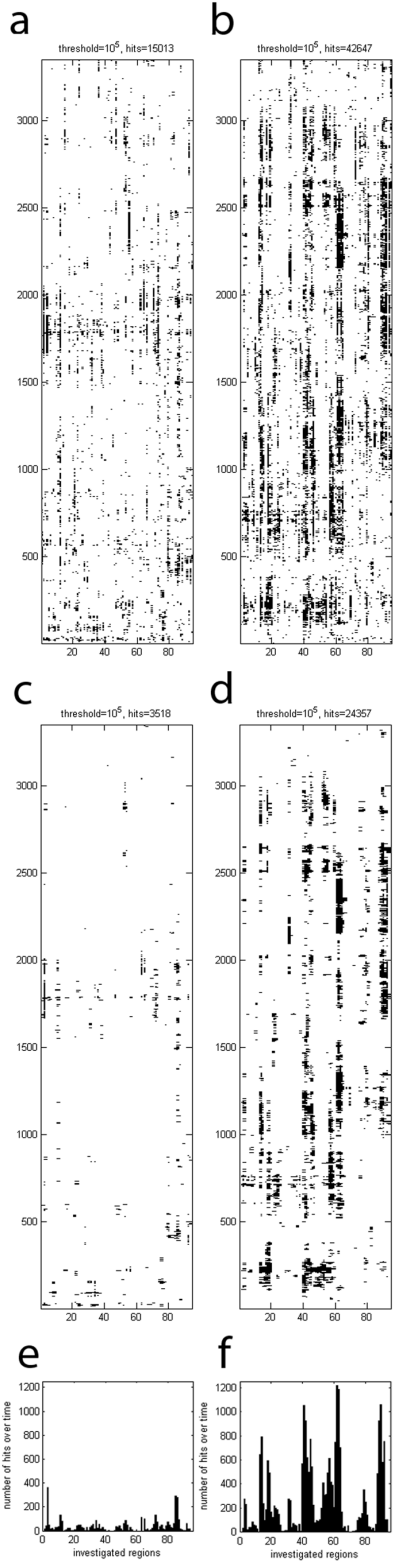
Images of differences for a true split and a typical random permutation split. The same methodology as in Figure 2 is applied. (A) Random permutation split number 413 without filters. This permutation has a total number of 3518 dots after the median filter. Since this value is almost the median number of dots (3517) over all 500 random permutation splits, permutation 413 was selected as a typical representative. (B) True split without filters. (C) The image of random permutation split 413 after median filter. (D) The image of the true split after application of the median filter. (E) Number of differences after median filter for random permutation split 413, as measured by the t-tests, for all regions as a sum over time. (F) Number of differences after median filter for the true split, as measured by the t-tests, for all regions as a sum over time.

In Figure 5 the corresponding difference image of one such random permutation split is depicted for comparison with the apparent systematic structure observed for the true split. The images depict the structure before (Figure 5AB) and after the median-method (Figure 5CD) to illustrate the effect of noise reduction. The systematicity value for the true split is clearly above the critical value (see also Figure 4). Therefore, we can speak of the observed image pattern as being a significant one. In comparison, the random permutation split illustrated in Figure 5 yielded a systematicity value which corresponds to the median systematicity value of all 500 random permutation splits.

### Most Frequently Different Regions

**Figure 6:**
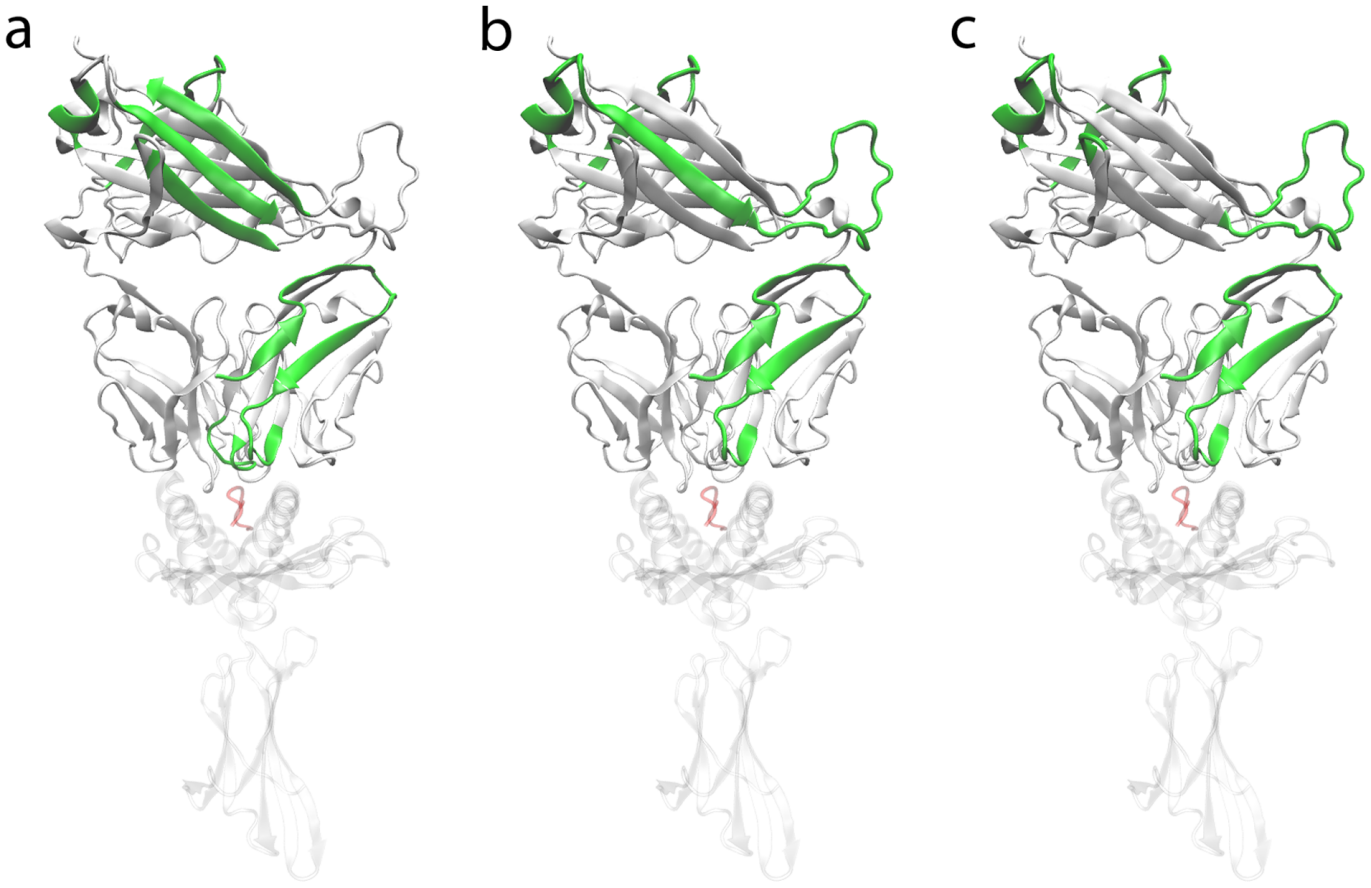
Illustration of the most frequently highlighted regions. White and solid: TCR. White and transparent: MHC. Red: peptide. Green: top 5 most frequently highlighted regions per threshold (i.e. 20 regions where several of them are identical). If the whole TCR beta chain was within the top regions it was not coloured in green for reasons of visibility. (A) Direct-method. (B) Median-method. (C) Square-method.

Among all 94 investigated regions in the TCR most regions with the largest number of differences over time form parts of the TCR beta chain. Out of the top 10 regions (the 10 regions with the highest amount of differences over time) for each of the 4 thresholds (i.e. 40 in total) 34 regions form parts of the TCR beta chain while 6 formparts of the TCR alpha chain (Figure 3 and Table 1). Among the top 20 regions over all 4 thresholds (i.e. 80 in total) 67 regions formparts of the TCR beta chain while 13 form parts of the TCR alpha chain. These findings are in agreement with Armstrong et al. who suggested that the beta-chain may play an important role in ligand recognition [32]. In Figure 6 the spatial arrangement of the top 5 regions per threshold and method are illustrated.

If we further investigate clusters of differences, as for example visible in the bar plots of Figure 3, the results point in the same direction. Clear clusters with high amounts of differences over time are visible. In most combinations of methods and thresholds the major differences are in and around the area of the CDRbeta regions. Additional clusters of differences are visible in and around regions 76 and 90 which are both part of the constant TCR beta chain.

This finding is consistent with the known immunological background that the recognition of the pMHC is carried out with the CDRs [1]. For the LC13 TCR in complex with HLA-B*08 detailed investigations on the CDR region were previously reported by Borg et al. [52]. Here our findings partly disagree with their conclusion that both CDR3 regions are the hotspot for the ‘energetic landscape’ of the pMHC recognition and that changes in the CDR1 and 2 are mainly used as stabilizer for the ligated CDR3s. However, it was also shown by other authors for different TCRpMHC systems that CDR1 and 2 can directly contribute [53].

It was previously reported that the A-B loop (residues 129 to 136) of the constant domain of the TCR alpha chain represents a “closed” conformation in the unbound state and switches to an “open” conformation upon ligation [54]. It was further reported that antagonistic ligands have a differential ability to change the conformation of the A-B loop. Using the median-method for the 94 investigated regions we found the A-B loop at rank 10, 25, 22, and 56 for our thresholds 1 to 4. The square-method yielded the ranks 8, 14, 17 and 30, while the direct method yielded 4, 21, 23, and 44. This observed sparse difference in the A-B loop may be caused by the different timescales on which MD simulations and the experimental techniques of Beddoe et al. work. However, it should be noted that the above described clusters of differences around the regions 76 and 90 are in close spatial proximity to the A-B loop.

Note, however, that our type of statistical testing only provides significance for the entirety of regions showing a systematic pattern (Figure 3) as defined in the direct- median- and square-methods. No immediate conclusions can be drawn for the statistical significance of single regions contained in this structure.

### Evaluation of 20 Trajectories with a Simulation Length of 50 Ns

As mentioned in the methods section we performed an additional set of simulations for a real time of 50 ns each. In this test set we used all possible substitutions of position 7 in the peptide yielding 20 trajectories of the LC13 TCR in complex with HLA-B*08∶01 presenting FLRGRAXGL (where X denotes for all 20 canonical amino acids).

**Figure 7:**
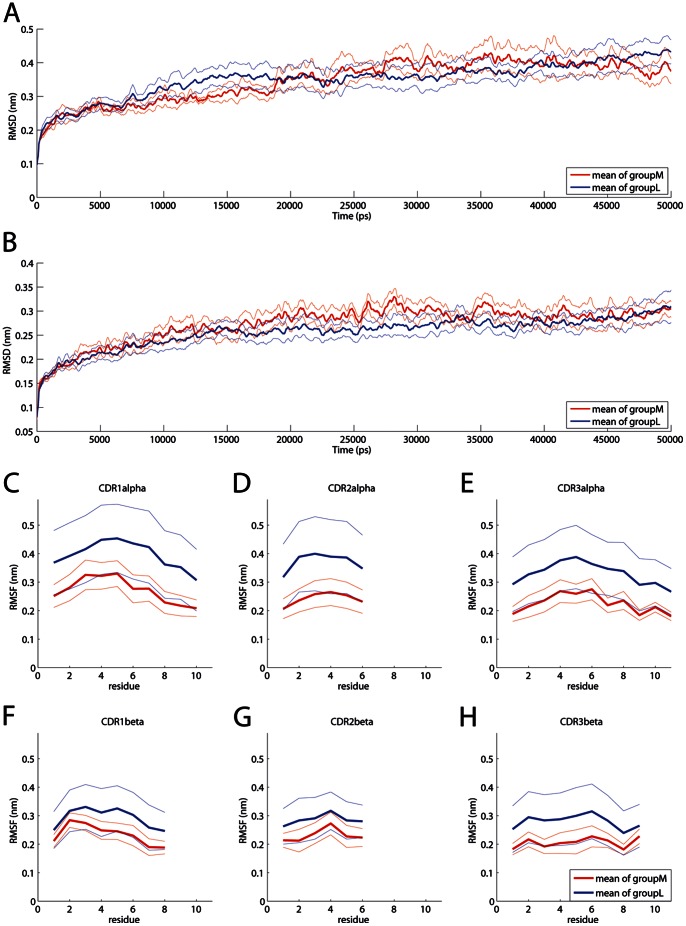
Comparison of groupM and groupL over a simulation time of 50 ns at threshold 10-^5^ M. For means of readability the curves of A and B were smoothed using a moving average. The mean value is indicated by a thick line while the mean value +/− the standard error of mean is indicated by thin lines. (A) RMSD TCR alpha chain. (B) RMSD TCR beta chain. (C-H) RMSF of the CDR regions.

Although there seems to be a tendency in the differences between the groups (Figure 7) the application of our above described methodology for only 20 simulations (14 groupM against 6 groupL at a threshold of 10^–5^ M) instead of 172 simulations did not yield statistically significant differences for the entirety of regions. However, this is an expectable result since the effect found in 172 trajectories was also not huge. Therefore it seems likely that 20 simulations are statistically underpowered for this type of analysis. Figure 7 shows the mean RMSD of the TCR alpha and beta chain for the 2 groups as well as the root mean square fluctuation (RMSF) of the CDRs. It can be seen that groupM and groupL differ in several time spans (Figure 7AB) as well as in their flexibility (Figure 7C–H).

### How Structural Dynamics Could Influence T Cell Triggering

Given the above described differences one could ask how these differences could further trigger T cell activation: There is plethora of different hypotheses for T cell triggering available (reviewed in [2]). In some models mechanical forces and accompanying deformations play a central role. For example Ma et al. propose that the T cell is activated by pulling forces originating from the cytoskeleton that induce conformational changes in the TCR/CD3 complex [55] [56]. In this model the weak binding between a TCR and a nonspecific pMHC can be ruptured without signalling while a specific pMHC bound to the same TCR triggers the T cell. By this way the TCR acts as an anisotropic mechanosensor [57]. The found differences in the spatial dynamics of the very early relaxation process between more and less immunogenic TCRpMHC complexes would be in line with the above described hypothesis that the TCR acts as a mechanosensor.

### Conclusion

MD simulations could provide ultimate details concerning individual particle motions for many aspects of biomolecular functions [58]. They range from the role of solvent in protein dynamics [59] via the description of the structural pathway between the open and closed conformation of GroEL [60], the flexibility of a short peptide linker governing the activation of tyrosine kinases [61], to opening and closing of the long channel in acetylcholinesterase [62], to protein folding [63].

In this study we applied MD to 172 closely related APLs of a well described TCRpMHC system. This approach gave indications that the very early relaxation dynamics differ between groups of TCRpMHC complexes with different levels of peptide immunogenicity. On this basis we conclude that there is a correlation between peptide immunogenicity and the way TCRpMHC complexes start their relaxation process from the initial (perturbed) x-ray structure within the first 10 ns of simulation. It was already shown that different physicochemical properties are related to peptide immunogenicity [9] and that structural rearrangement during TCR binding contributes to T cell activation [6]. On this basis it is consequential that different physicochemical properties cause different structural dynamics and could be another level of T cell regulation. However, we also want to point out that the shortness of the simulations could have introduced a systematic bias, and large conformational changes taking place may not have been sampled by these simulations. Additionally an evaluation of a subset of 20 simulations with a length of 50 ns did show differences between the groups, however, they were not statistically significant, which might be caused by the too small sample size. Hence, further studies with several hundred of complexes with several hundred of nanoseconds each will be necessary to finally prove or disprove differences between more and less immunogenic TCRpMHC complexes.

It is known that the variable loops of the TCR undergo significant changes upon pMHC engagement leading to a loss of flexibility in the loops [64]. On this basis our study gives indications that these changes might differ on the basis of different peptide immunogenicity levels. Thereby our findings agree well with the proposed idea that the TCR functions as a mechanosensor [57] and the beta-chain may play an important role in ligand recognition [32]. On this basis our findings might have implications on the development of predictive methods, since the number of methods directly predicting immunogenicity of pMHC is limited (see Introduction).

## References

[1] Rudolph MG, Stanfield RL, Wilson IA (2006) How TCRs bind MHCs, peptides, and coreceptors. Annu Rev Immunol 24: 419–466. 16551255 10.1146/annurev.immunol.23.021704.115658

[2] Choudhuri K, van der Merwe PA (2007) Molecular mechanisms involved in T cell receptor triggering. Semin Immunol 19: 255–261. 17560121 10.1016/j.smim.2007.04.005

[3] Wucherpfennig KW, Allen PM, Celada F, Cohen IR, De BR, et al (2007) Polyspecificity of T cell and B cell receptor recognition. Semin Immunol 19: 216–224. 17398114 10.1016/j.smim.2007.02.012 PMC2034306

[4] Hemmer B, Vergelli M, Gran B, Ling N, Conlon P, et al (1998) Predictable TCR antigen recognition based on peptide scans leads to the identification of agonist ligands with no sequence homology. J Immunol 160: 3631–3636. 9558061

[5] Guy CS, Vignali DA (2009) Organization of proximal signal initiation at the TCR:CD3 complex. Immunol Rev 232: 7–21. 19909352 10.1111/j.1600-065X.2009.00843.x PMC2845712

[6] Krogsgaard M, Prado N, Adams EJ, He XL, Chow DC, et al (2003) Evidence that structural rearrangements and/or flexibility during TCR binding can contribute to T cell activation. Mol Cell 12: 1367–1378. 14690592 10.1016/s1097-2765(03)00474-x

[7] Frankild S, de Boer RJ, Lund O, Nielsen M, Kesmir C (2008) Amino acid similarity accounts for T cell cross-reactivity and for “holes” in the T cell repertoire. PLoS ONE 3: e1831. 18350167 10.1371/journal.pone.0001831 PMC2263130

[8] Tung CW, Ziehm M, Kamper A, Kohlbacher O, Ho SY (2011) POPISK: T-cell reactivity prediction using support vector machines and string kernels. BMC Bioinformatics 12: 446. 22085524 10.1186/1471-2105-12-446 PMC3228774

[9] Tung CW, Ho SY (2007) POPI: predicting immunogenicity of MHC class I binding peptides by mining informative physicochemical properties. Bioinformatics 23: 942–949. 17384427 10.1093/bioinformatics/btm061

[10] Myers EW, Miller W (1988) Optimal alignments in linear space. Comput Appl Biosci 4: 11–17. 3382986 10.1093/bioinformatics/4.1.11

[11] Altschul SF, Madden TL, Schaffer AA, Zhang J, Zhang Z, et al (1997) Gapped BLAST and PSI-BLAST: a new generation of protein database search programs. Nucleic Acids Res 25: 3389–3402. 9254694 10.1093/nar/25.17.3389 PMC146917

[12] Tong JC, Tan TW, Ranganathan S (2007) Methods and protocols for prediction of immunogenic epitopes. Brief Bioinform 8: 96–108. 17077136 10.1093/bib/bbl038

[13] Mishra S, Sinha S (2009) Immunoinformatics and modeling perspective of T cell epitope-based cancer immunotherapy: a holistic picture. J Biomol Struct Dyn 27: 293–306. 19795913 10.1080/07391102.2009.10507317

[14] Yewdell JW, Bennink JR (1999) Immunodominance in major histocompatibility complex class I-restricted T lymphocyte responses. Annu Rev Immunol 17: 51–88. 10358753 10.1146/annurev.immunol.17.1.51

[15] Hansson T, Oostenbrink C, Van Gunsteren WF (2002) Molecular dynamics simulations. Curr Opin Struct Biol 12: 190–196. 11959496 10.1016/s0959-440x(02)00308-1

[16] Cuendet MA, Michielin O (2008) Protein-protein interaction investigated by steered molecular dynamics: the TCR-pMHC complex. Biophys J 95: 3575–3590. 18621828 10.1529/biophysj.108.131383 PMC2553100

[17] Yaneva R, Springer S, Zacharias M (2009) Flexibility of the MHC class II peptide binding cleft in the bound, partially filled, and empty states: A molecular dynamics simulation study. Biopolymers 91: 14–27. 18767126 10.1002/bip.21078

[18] Wan S, Coveney PV, Flower DR (2005) Molecular basis of peptide recognition by the TCR: affinity differences calculated using large scale computing. J Immunol 175: 1715–1723. 16034112 10.4049/jimmunol.175.3.1715

[19] Painter CA, Cruz A, Lopez GE, Stern LJ, Zavala-Ruiz Z (2008) Model for the peptide-free conformation of class II MHC proteins. PLoS ONE 3: e2403. 18545669 10.1371/journal.pone.0002403 PMC2408972

[20] Zacharias M, Springer S (2004) Conformational flexibility of the MHC class I alpha1-alpha2 domain in peptide bound and free states: a molecular dynamics simulation study. Biophys J 87: 2203–2214. 15454423 10.1529/biophysj.104.044743 PMC1304646

[21] Wan S, Flower DR, Coveney PV (2008) Toward an atomistic understanding of the immune synapse: Large-scale molecular dynamics simulation of a membrane-embedded TCR-pMHC-CD4 complex. Molecular Immunology 45: 1221–1230. 17980430 10.1016/j.molimm.2007.09.022

[22] Rognan D, Stryhn A, Fugger L, Lyngbaek S, Engberg J, et al (2000) Modeling the interactions of a peptide-major histocompatibility class I ligand with its receptors. I. Recognition by two alpha beta T cell receptors. J Comput Aided Mol Des 14: 53–69. 10702925 10.1023/a:1008142830353

[23] Haidar JN, Pierce B, Yu Y, Tong W, Li M, et al (2009) Structure-based design of a T-cell receptor leads to nearly 100-fold improvement in binding affinity for pepMHC. Proteins 74: 948–960. 18767161 10.1002/prot.22203 PMC2696811

[24] Camacho CJ, Katsumata Y, Ascherman DP (2008) Structural and thermodynamic approach to peptide immunogenicity. PLoS Comput Biol 4: e1000231. 19023401 10.1371/journal.pcbi.1000231 PMC2577884

[25] De Rosa MC, Giardina B, Bianchi C, Carelli AC, Pirolli D, et al (2010) Modeling the ternary complex TCR-Vbeta/CollagenII(261–273)/HLA-DR4 associated with rheumatoid arthritis. PLoS ONE 5: e11550. 20644721 10.1371/journal.pone.0011550 PMC2904365

[26] Stavrakoudis A (2011) Insights into the structure of the LC13 TCR/HLA-B8-EBV peptide complex with molecular dynamics simulations. Cell Biochem Biophys 60: 283–295. 21253892 10.1007/s12013-011-9151-2

[27] Cuendet MA, Zoete V, Michielin O (2011) How T cell receptors interact with peptide-MHCs: a multiple steered molecular dynamics study. Proteins 79: 3007–3024. 21989928 10.1002/prot.23104

[28] Narzi D, Becker CM, Fiorillo MT, Uchanska-Ziegler B, Ziegler A, et al (2012) Dynamical characterization of two differentially disease associated MHC class I proteins in complex with viral and self-peptides. J Mol Biol 415: 429–442. 22119720 10.1016/j.jmb.2011.11.021

[29] Knapp B, Omasits U, Bohle B, Maillere B, Ebner C, et al (2009) 3-Layer-based analysis of peptide-MHC-interaction: in silico prediction, peptide binding affinity and T cell activation in a relevant allergen-specific model. Molecular Immunology 46: 1839–1844. 19232439 10.1016/j.molimm.2009.01.009

[30] Knapp B, Fischer G, Van HD, Fae I, Maillere B, et al (2012) Association of HLA-DR1 with the allergic response to the major mugwort pollen allergen: molecular background. BMC Immunol 13: 43. 22871092 10.1186/1471-2172-13-43 PMC3522052

[31] Knapp B, Omasits U, Schreiner W, Epstein MM (2010) A comparative approach linking molecular dynamics of altered peptide ligands and MHC with in vivo immune responses. PLoS ONE 5: e11653. 20657836 10.1371/journal.pone.0011653 PMC2906508

[32] Armstrong KM, Piepenbrink KH, Baker BM (2008) Conformational changes and flexibility in T-cell receptor recognition of peptide-MHC complexes. Biochem J 415: 183–196. 18800968 10.1042/BJ20080850 PMC2782316

[33] Bergman HM, Westbrook J, Feng Z, Gilliland G, Bhat TN, et al (2000) The Protein Data Bank. Nucleic Acids Res 28: 235–242. 10592235 10.1093/nar/28.1.235 PMC102472

[34] Kjer-Nielsen L, Clements CS, Purcell AW, Brooks AG, Whisstock JC, et al (2003) A structural basis for the selection of dominant alphabeta T cell receptors in antiviral immunity. Immunity 18: 53–64. 12530975 10.1016/s1074-7613(02)00513-7

[35] Kjer-Nielsen L, Clements CS, Brooks AG, Purcell AW, McCluskey J, et al (2002) The 1.5 A crystal structure of a highly selected antiviral T cell receptor provides evidence for a structural basis of immunodominance. Structure 10: 1521–1532. 12429093 10.1016/s0969-2126(02)00878-x

[36] Kjer-Nielsen L, Clements CS, Brooks AG, Purcell AW, Fontes MR, et al (2002) The structure of HLA-B8 complexed to an immunodominant viral determinant: peptide-induced conformational changes and a mode of MHC class I dimerization. J Immunol 169: 5153–5160. 12391232 10.4049/jimmunol.169.9.5153

[37] Burrows SR, Silins SL, Cross SM, Peh CA, Rischmueller M, et al (1997) Human leukocyte antigen phenotype imposes complex constraints on the antigen-specific cytotoxic T lymphocyte repertoire. Eur J Immunol 27: 178–182. 9022015 10.1002/eji.1830270126

[38] Rognan D, Zimmermann N, Jung G, Folkers G (1992) Molecular dynamics study of a complex between the human histocompatibility antigen HLA-A2 and the IMP58–66 nonapeptide from influenza virus matrix protein. Eur J Biochem 208: 101–113. 1511679 10.1111/j.1432-1033.1992.tb17163.x

[39] Zoete V, Michielin O (2007) Comparison between computational alanine scanning and per-residue binding free energy decomposition for protein-protein association using MM-GBSA: Application to the TCR-p-MHC complex. Proteins 67: 1026–1047. 17377991 10.1002/prot.21395

[40] Gregoire C, Lin SY, Mazza G, Rebai N, Luescher IF, et al (1996) Covalent assembly of a soluble T cell receptor-peptide-major histocompatibility class I complex. Proc Natl Acad Sci U S A 93: 7184–7189. 8692966 10.1073/pnas.93.14.7184 PMC38957

[41] Toh H, Kamikawaji N, Tana T, Muta S, Sasazuki T, et al (2000) Magnitude of structural changes of the T-cell receptor binding regions determine the strength of T-cell antagonism: molecular dynamics simulations of HLA-DR4 (DRB1*0405) complexed with analogue peptide. Protein Eng 13: 423–429. 10877853 10.1093/protein/13.6.423

[42] Omasits U, Knapp B, Neumann M, Steinhauser O, Stockinger H, et al (2008) Analysis of Key Parameters for Molecular Dynamics of pMHC Molecules. Mol Simulat 34: 781–793.

[43] Wan S, Coveney PV, Flower DR (2004) Large-scale molecular dynamics simulations of HLA-A*0201 complexed with a tumor-specific antigenic peptide: can the alpha3 and beta2m domains be neglected? J Comput Chem 25: 1803–1813. 15386470 10.1002/jcc.20100

[44] Canutescu AA, Shelenkov AA, Dunbrack RL (2003) A graph-theory algorithm for rapid protein side-chain prediction. Protein Sci 12: 2001–2014. 12930999 10.1110/ps.03154503 PMC2323997

[45] Guex N, Peitsch MC (1997) SWISS-MODEL and the Swiss-PdbViewer: an environment for comparative protein modeling. Electrophoresis 18: 2714–2723. 9504803 10.1002/elps.1150181505

[46] Knapp B, Omasits U, Schreiner W (2008) Side chain substitution benchmark for peptide/MHC interaction. Protein Sci 17: 977–982. 18434501 10.1110/ps.073402508 PMC2386744

[47] Knapp B, Omasits U, Frantal S, Schreiner W (2009) A critical cross-validation of high throughput structural binding prediction methods for pMHC. J Comput Aided Mol Des 23: 301–307. 19194661 10.1007/s10822-009-9259-2

[48] Hess B, Kutzner C, vanderSpoel D, Lindahl E (2008) GROMACS 4: Algorithms for Highly Efficient, Load-Balanced, and Scalable Molecular Simulation. J Chem Theory Comput 4: 435–447. 26620784 10.1021/ct700301q

[49] Mazur AK (1998) Hierarchy of Fast Motions in Protein Dynamics. J Phys Chem B 102: 473–479.

[50] Feenstra KA, Hess B, Berendsen HJ (1999) Improving efficiency of large time-scale molecular dynamics simulations of hydrogen-rich systems. J Comput Chem 768–798. 10.1002/(SICI)1096-987X(199906)20:8<786::AID-JCC5>3.0.CO;2-B 35619462

[51] Humphrey W, Dalke A, Schulten K (1996) VMD: visual molecular dynamics. J Mol Graph 14: 33–38. 8744570 10.1016/0263-7855(96)00018-5

[52] Borg NA, Ely LK, Beddoe T, Macdonald WA, Reid HH, et al (2005) The CDR3 regions of an immunodominant T cell receptor dictate the 'energetic landscape' of peptide-MHC recognition. Nat Immunol 6: 171–180. 15640805 10.1038/ni1155

[53] Manning TC, Schlueter CJ, Brodnicki TC, Parke EA, Speir JA, et al (1998) Alanine scanning mutagenesis of an alphabeta T cell receptor: mapping the energy of antigen recognition. Immunity 8: 413–425. 9586632 10.1016/s1074-7613(00)80547-6

[54] Beddoe T, Chen Z, Clements CS, Ely LK, Bushell SR, et al (2009) Antigen ligation triggers a conformational change within the constant domain of the alphabeta T cell receptor. Immunity 30: 777–788. 19464197 10.1016/j.immuni.2009.03.018

[55] Ma Z, Janmey PA, Finkel TH (2008) The receptor deformation model of TCR triggering. FASEB J 22: 1002–1008. 17984179 10.1096/fj.07-9331hyp PMC2679516

[56] Ma Z, Finkel TH (2010) T cell receptor triggering by force. Trends Immunol 31: 1–6. 19836999 10.1016/j.it.2009.09.008 PMC2818226

[57] Kim ST, Takeuchi K, Sun ZY, Touma M, Castro CE, et al (2009) The alphabeta T cell receptor is an anisotropic mechanosensor. J Biol Chem 284: 31028–31037. 19755427 10.1074/jbc.M109.052712 PMC2781503

[58] Karplus M, McCammon JA (2002) Molecular dynamics simulations of biomolecules. Nat Struct Biol 9: 646–652. 12198485 10.1038/nsb0902-646

[59] Vitkup D, Ringe D, Petsko GA, Karplus M (2000) Solvent mobility and the protein 'glass' transition. Nat Struct Biol 7: 34–38. 10625424 10.1038/71231

[60] Ma J, Sigler PB, Xu Z, Karplus M (2000) A dynamic model for the allosteric mechanism of GroEL. J Mol Biol 302: 303–313. 10970735 10.1006/jmbi.2000.4014

[61] Young MA, Gonfloni S, Superti-Furga G, Roux B, Kuriyan J (2001) Dynamic coupling between the SH2 and SH3 domains of c-Src and Hck underlies their inactivation by C-terminal tyrosine phosphorylation. Cell 105: 115–126. 11301007 10.1016/s0092-8674(01)00301-4

[62] Tai K, Shen T, Borjesson U, Philippopoulos M, McCammon JA (2001) Analysis of a 10-ns molecular dynamics simulation of mouse acetylcholinesterase. Biophys J 81: 715–724. 11463620 10.1016/S0006-3495(01)75736-0 PMC1301548

[63] Shaw DE, Maragakis P, Lindorff-Larsen K, Piana S, Dror RO, et al (2010) Atomic-level characterization of the structural dynamics of proteins. Science 330: 341–346. 20947758 10.1126/science.1187409

[64] Schamel WW, Reth M (2007) The TCR binding site does move. Proc Natl Acad Sci U S A 104: 16398–16399. 17925433 10.1073/pnas.0708462104 PMC2034258

